# On the Use of Electrooculogram for Efficient Human Computer Interfaces

**DOI:** 10.1155/2010/135629

**Published:** 2009-10-15

**Authors:** A. B. Usakli, S. Gurkan, F. Aloise, G. Vecchiato, F. Babiloni

**Affiliations:** ^1^IRCCS Fondazione Santa Lucia, Via Ardeatina, 306, 00179, Rome, Italy; ^2^Department of Technical Sciences, The NCO Academy, 10100 Balikesir, Turkey

## Abstract

The aim of this study is to present electrooculogram signals that can be used for human computer interface efficiently. Establishing an efficient alternative channel for communication without overt speech and hand movements is important to increase the quality of life for patients suffering from Amyotrophic Lateral Sclerosis or other illnesses that prevent correct limb and facial muscular responses. We have made several experiments to compare the P300-based BCI speller and EOG-based new system. A five-letter word can be written on average in 25 seconds and in 105 seconds with the EEG-based device. Giving message such as “clean-up” could be performed in 3 seconds with the new system. The new system is more efficient than P300-based BCI system in terms of accuracy, speed, applicability, and cost efficiency. Using EOG signals, it is possible to improve the communication abilities of those patients who can move their eyes.

## 1. Introduction

An efficient alternative channel for communication without speech and hand movements is important to increase the quality of life for patients suffering from Amyotrophic Lateral Sclerosis or other illnesses that prevent correct limb and facial muscular responses. In this respect, the area of study related to the Human Computer Interaction and Brain Computer Interface (BCI) is very important in hopes of improving the medium term quality of the life for such patients.

In eye movements, a potential across the cornea and retina exists, and it is source of electrooculogram (EOG). EOG can be modeled by a dipole [1], and these systems can be used in medical systems. There are several EOG-based HCI studies in literature. A wheelchair controlled with eye movements is developed for the disabled and elderly people. Eye movement signals and sensor signals are combined, and both direction and acceleration are controlled [2]. Using horizontal and vertical eye movements and two and three blinking signals a movable robot is controlled [3]. Because the EOG signals are slightly different for the each subject, a dynamical threshold algorithm is developed [4]. In this approach, the initial threshold is compared with the dynamic range; the threshold value is renewed after each difference. According to this threshold the output signal is made 1 or 0 and afterwards it is processed. EOG, EEG and electromyogram (EMG) signals are classified in real time, and movable robots are controlled by using artificial neural network classifier [5, 6]. Investigating possibility of usage of the EOG for HCI, a relation between sight angle and EOG is determined [7].

The human-machine interface which provides control of machines for disabled people is called the Man Machine Interface (MMI). Generally, if the control is computer-based, it is called the Human Computer Interface (HCI) instead of MMI. If the assistive system is based on electroencephalogram (EEG), it is called BCI, and its applications are increasing for severely disabled people. BCI is a direct communication pathway between the brain and an external device. The BCI systems translate brain activity into electrical signals that control external devices. Thus they can represent the only technology for severely paralyzed patients to increase or maintain their communication and control options [8]. Because EEG signals are characterized by low amplitude (*μ*V), their measurement is more difficult than EOG.

As a contribution in this area of research, in this study, we present an HCI device that is able to recognize the subject's eye movements by using the collection of the electrical activity generated by the eye, that is, EOG. This device allows the patients to generate decisions on a screen by means of simple eye movements and the electroencephalogram (EEG) electrodes, without the need of sophisticated infrared cameras. Then, patients could be able to select letters on the screen, or even to communicate basic needs (food, drinks, etc.) to the caregiver with a simple movement of their eyes.

In this study, to make experimental comparison, we made two experiments with the BCI system and realized EOG system (the design rationale presented in [9]). For each device the subjects wanted to write a five-letter word. The performance of the EOG system is relatively good, since a five-letter word can be written by the patient on average in 25 seconds and in 105 seconds with the EEG-based device. Giving message such as “clean up” could be performed in 3 seconds. The experiments' details are presented in the experimental results.

The paper is organized as follows: first, the new EOG-based HCI device will be presented. In this section the design is detail is briefly explained. Successively, experimental results will be illustrated.

## 2. Materials and Methods

### 2.1. The New EOG-Based HCI Device

**Figure 1:**
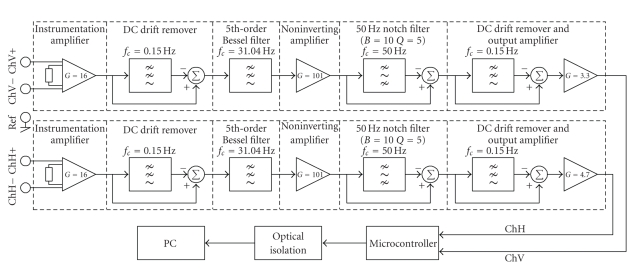
The new EOG system block diagram.

**Figure 2:**
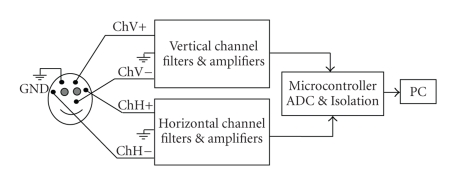
The EOG electrode configuration.

In this subsection, as an HCI device, a novel EOG measurement system design is proposed. Horizontal and vertical eye movements are measured with two passive electrodes usually employed for the EEG acquisition. The system block diagram is presented in Figure 1, and its electrode configuration in Figure 2. The system is microcontroller-based and battery powered. The CMRR is 88 dB, electronic noise is 0.6 *μ*V (*p*-*p*), and sampling rate is 176 Hz. 5 Ag/AgCl electrodes are used (two for each channel and one is for ground). In order to remove the DC level and 50 Hz power line noise, the differentiate approach is used. This approach is much more successful than classical methods.

### 2.2. The Design Details

**Figure 3:**
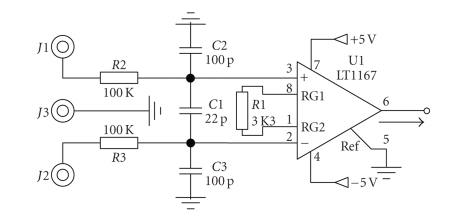
Input amplifier circuit.

After filtering and the amplification stages, the EOG signals are digitized (10 bit) and then transferred to the PC. The EOG signals are then processed by a classification algorithm which is based on the nearest neighborhood (NN) relation, with a classification performance of 95%. The EOG measurement system, as an HCI, allows people to communicate with their environment, only by using eye movements, successfully and economically (180 USD). The system's initial electronic circuitry (Figure 3) can be used for EOG, EMG, and EEG. After digitizing, horizontal and vertical EOG signals are then transferred to the PC serial port. Microcode Studio program is used to write the embedded code; Winpic800 is used to program the microcontroller (*μ*C). The data transfer rate is enough for the sampling rate (176 Hz), which is sufficient to process the EOG signals.

It is preferred to use the NN algorithm to classify the EOG signals since in this way they can be easily discriminated. The time cost of this algorithm is shorter than the other, more complex, classification ones. Regarding the NN, the Euclidean distance formula is used as follows:

(1) $$L\left(x,y\right)=\sqrt{\overset{d}{\underset{i=1}{\sum }}{\left(x_{i}-y_{i}\right)}^{2}}.$$

As for the classification, 5 classes (each having 20 members) are used. Each member consists of 251 samples. To increase the classification performance, both channels are applied together to the classifier. The classification performance is 95%. System software transfers the data and classifies it in real time.

**Figure 4:**
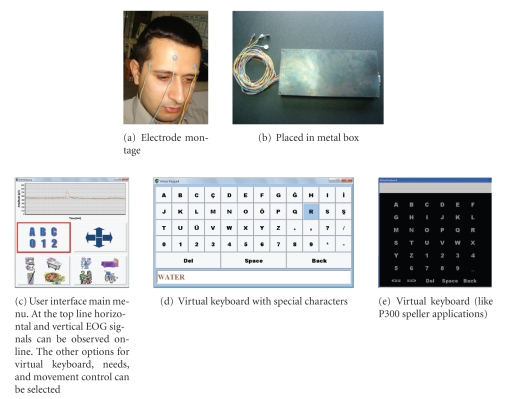
The realized EOG-based device.

Summarizing, the realized system (Figure 4) is based on the following features:

- horizontal and vertical eye movement signals are acquired,
- Ag/AgCl electrodes are used,
- DC level and power line noise signals are removed with a subtraction approach,
- *μ*C-based,
- battery powered,
- the NN algorithm is used for the classification,
- user-friendly interface.

## 3. Experimental Results

To compare the systems we made experiments with the BCI system (8 channels, Guger Technology) and new EOG system. The P300-based BCI speller based on the detection of P300 waveforms from the array of 8 electrodes returned in 21 seconds (105 seconds for 5 letters) for the selection of the word “water” in the experimental group (10 subjects) employed. In addition, the accuracy of the letter selection on average was 81% in the same group, with a standard deviation of 14%. Controls were then able to master the P300 BCI system after a session of 30 minutes at the reported level of accuracy.

The group of 10 subjects that selected the word “water” with the EOG-based device employed an average time of 24.7 seconds, with 3.2 seconds as a standard deviation. The accuracy percentage in this group was 100%, regarding writing of the selected word. Also in this case the subjects were able to master the device after a session of 5 minutes. Notifying a need message (clean up) could be performed in 3 seconds.

**Figure 5:**
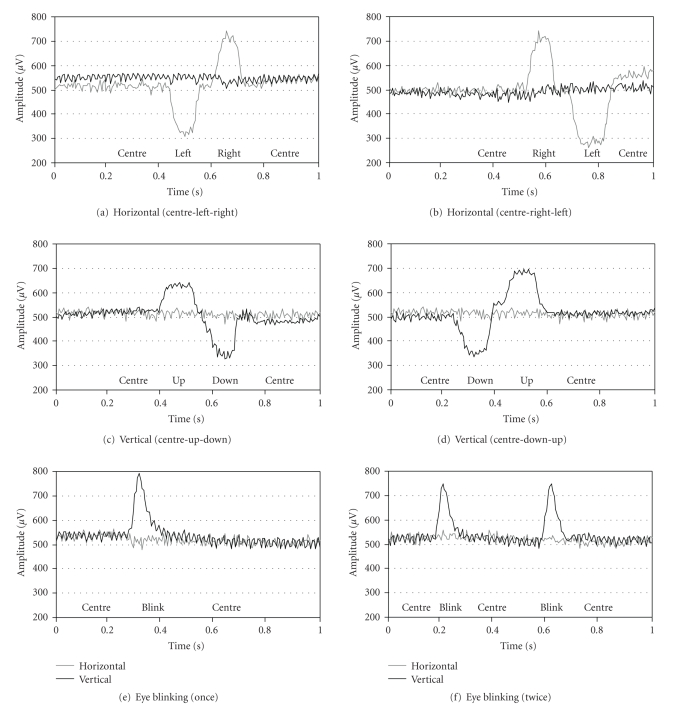
The EOG recording samples.

As seen from the recordings in Figure 5, after considering noise reduction measures in designing of the biopotential data acquisition system, the EOG system performance is good. Electronic noise reduction is also successful. The circuit can be easily adapted for EMG and EEG measurements.

## 4. Conclusion

In this paper we proposed a new system to use the EOG signals for the realization of an HCI device able to restore some communication abilities to patients not able to move their limbs and facial muscles. After our experiments, it is observed that the new EOG-based system can be used for HCI, efficiently. From a technical point of view the highlights of the presented system are the following.

- Horizontal and vertical EOG signals are measured successfully. CMRR is 88 dB, sampling rate is 176 Hz, and electronic noise is 0.6 *μ*V (*p*-*p*). According to the specifications, the present system can measure the EOG signals properly.
- The EOG signals, for different eye movements, are classified on-line. The NN algorithm (with Euclidean distance) is used. The signals do not need complex and time-costly classification algorithms.
- The realized virtual keyboard allows the user to write messages and to communicate other needs relatively in an efficient way.

The EOG-based system seems more efficient than EEG-based (P300 BCI). It must be noted that the solution for the EOG system is extremely cheap when compared to the EEG solution (one order of magnitude) and then can be used as a first step for the hybrid device for the final users. A hybrid device is to familiarize the patient with a unique interface while he/she could switch with the biosignal more useful for him/her in that particular moment in time for the communication or for the control of the external devices. In this respect there will be the possibility to change the control signals without the need to relearn the user interfaces, as usually happen today with the use of different interfaces.

The realized system will be now tested by several patients in order to improve the quality of the graphic interface for a better and quick selection of the interesting items by using the EOG signals. As a future work, our research group will investigate using combined EOG and EEG and other inputs [10–15] for efficient configuration of a multi-input hybrid HCI.

## References

[1] Venkataramanan S, Prabhat P, Choudhury SR, Nemade HB, Sahambi JS Biomedical instrumentation based on Electrooculogram (EOG) signal processing and application to a hospital alarm system.

[2] Barae R, Boquete L, Mazo M (2002). System for assited mobility using eye movements based on electrooculography. *IEEE Transaction on Neural Systems and Rehabilitation Engineering*.

[3] Kim Y, Doh NL, Youm Y, Chung WK (2007). Robust discrimination method of the electrooculogram signals for human-computer interaction controlling mobile robot. *Intelligent Automation and Soft Computing*.

[4] Lv Z, Wu X, Li M, Zhang C Implementation of the EOG-based human computer interface system.

[5] Young CK, Sasaki M Mobile robot control by neural network EOG gesture recognition.

[6] Chen Y, Newman WS A human-robot interface based on electrooculography.

[7] Kumar D, Poole E Classification of EOG for human computer interface.

[8] Cincotti F, Mattia D, Aloise F (2008). Non-invasive brain-computer interface system: towards its application as assistive technology. *Brain Research Bulletin*.

[9] Usakli AB, Gurkan S Design of a novel efficient human computer interface: an electrooculagram based virtual keyboard.

[10] De Vico Fallani F, Astolfi L, Cincotti F (2007). Cortical Functional Connectivity Networks In Normal And Spinal Cord Injured
Patients: Evaluation by Graph Analysis. *Human Brain Mapping*.

[11] Astolfi L, De Vico Fallani F, Cincotti F (2007). Imaging Functional Brain Connectivity Patterns From High-Resolution EEG And fMRI Via Graph Theory. *Psychophysology*.

[12] Astolfi L, Cincotti F, Mattia D (2008). Tracking the time-varying cortical connectivity patterns by adaptive multivariate estimators. *IEEE Trans on Biomedical Engineering*.

[13] Oliveri M, Babiloni C, Filippi MM (2003). Influence of the supplementary motor area on primary motor cortex excitability during
movements triggered by neutral or emotionally unpleasant visual cues. *Exp Brain Res*.

[14] Babiloni C, Babiloni F, Carducci F (2001). Mapping of early and late human somatosensory evoked brain potentials to phasic galvanic painful
stimulation. *Human Brain Mapping*.

[15] Urbano A, Babiloni C, Carducci F, Fattorini L, Onorati P, Babiloni F (1998). Dynamic functional coupling of high resolution EEG potentials
related to unilateral internally triggered one-digit movements. *Electroencephalography and clinical Neurophysiol*.

